# The impact of out-of-pocket payments for dental care on household finances in low and middle income countries

**DOI:** 10.1186/s12889-017-4042-0

**Published:** 2017-01-23

**Authors:** Eduardo Bernabé, Mohd Masood, Marko Vujicic

**Affiliations:** 1 Division of Population and Patient Health, King’s College London Dental Institute at Guy’s, King’s College and St. Thomas’ Hospitals, Denmark Hill Campus, Bessemer Road, London, SE5 9RS UK; 2 Department of Dentistry and Oral Health, La Trobe University, Melbourne, Australia; 3 Health Policy Institute, American Dental Association, Chicago, USA

**Keywords:** Cost of illness, Dental care, Developing countries, Multilevel analysis

## Abstract

**Background:**

Dental care is extremely costly and beyond most people means in developing countries. The primary aim of this study was to determine the impact of out-of-pocket payments for dental care on household finances in 40 low and middle income countries. A second aim was to compare the burden of payments for dental care with that for other health services.

**Methods:**

We used data from 174,257 adults, aged 18 years and above, who reported their total and itemized household expenditure in the past four weeks as part of the World Health Surveys. The financial burden on households was measured using the catastrophic health expenditure (CHE) and impoverishment approaches. A household was classified as facing CHE if it spent 40% or more of its capacity to pay, and as facing impoverishment if it fell below the country-specific poverty line after spending on health care was subtracted from household expenditure. The odds of experiencing CHE and impoverishment due to expenditure on dental care were estimated from two-level logistic regression models, controlling for various individual- and country-level covariates.

**Results:**

Households that paid for dental care had 1.88 (95% Confidence Interval: 1.78-1.99) greater odds of incurring CHE and 1.65 (95% CI: 1.52–1.80) greater odds of facing impoverishment, after adjustment for covariates. Furthermore, the impact of paying for dental care was lower than that for medications or drugs, inpatient care, outpatient care and laboratory tests but similar to that of health care products, traditional medicine and other health services.

**Conclusion:**

Households with recent dental care spending were more likely to use a large portion of their disposable income and fall below the poverty line. Policy makers ought to consider including dental care as part of universal health care and advocate for the inclusion of dental care coverage in health insurance packages.

## Background

Governments around the world have been called on to move towards universal health coverage for their citizens [1]. Such commitment requires that everyone receives needed health care services without experiencing financial difficulty [1, 2]. Despite recent efforts to search for alternative health financing mechanisms [3, 4], out-of-pocket payments are the primary mechanism to finance health services in low and middle income countries [2, 5]. Large and unpredictable out-of-pocket expenses for health care services may push families to spend considerable proportions of their disposable income (also known as catastrophic health expenditure or CHE) and, at the most extreme, push households into poverty (also known as impoverishment) [6–8].

There is evidence showing that certain countries and households are more likely to face financial hardship. Poorer and more unequal countries are more likely to have more households facing CHE [9, 10]. In addition, households that are in rural areas, low income, have older adults, young children or disabled members, and lack health insurance are more likely to face CHE and impoverishment [11–13]. The use of specific health services, such as inpatient care, prescription drugs and even visits to traditional healers, may also lead to CHE and impoverishment [14–17].

Millions of people worldwide suffer from oral diseases [18–20]. A total of US$298 billion are spent worldwide every year to cover the direct treatment costs associated with common oral conditions; a figure that represents 4.6% of health expenditure globally [21]. Most dental services are provided by private dentists to patients and are financed and delivered largely separate from medical services [22]. Out-of-pocket expenses make up a significant proportion of total dental expenditure, even in countries with high private dental insurance coverage like the Unites States [23]. These features make dental care extremely costly and beyond most people’s means in developing countries. A recent multilevel study across 41 developing countries showed that up to 6.8% of households incurred dental care expenditure in the past four weeks that was equal or greater than 40% of their capacity to pay, the so-called catastrophic dental health expenditure [24]. The problem with such an approach (i.e. including dental care rather than *any* healthcare expenditure in the numerator for the calculation) is that it does not allow comparison of the relative contribution of different types of health care services to financial burden. For that, one needs to see whether households that paid for specific health services are more likely to face financial hardship defined using standard metrics such as CHE and impoverishment. Two early studies suggest dental care expenditure may be a key contributor to CHE. In South Korea, the proportion of CHE was higher in households that used dental services (24.6%) than in those that did not use them (7.8%), although other determinants of CHE were not accounted for during the analysis [15]. In Iran, households that used dental services were, on average, four times more likely to incur CHE than those that did not use dental services [16]. The impact of payments for dental care on impoverishment has not been formally assessed.

The primary aim of this study was to determine the impact of out-of-pocket payments for dental care on household finances in 40 low and middle income countries. A second aim was to compare the burden of payments for dental care with that for other health services.

## Methods

### Data source

Individual-level data from the World Health Survey (WHS), carried out by the World Health Organization (WHO) in 2002–2004, were merged with country-level data from different international sources. The WHS aimed to provide valid, reliable, and comparable information from 70 participating countries regarding health status and health systems [25]. WHS data have been used frequently for the purpose of descriptive and analytical epidemiological investigations [26–28]. The study population was adults aged 18 years or older in private households in every country, who were recruited using multistage stratified cluster sampling. However, the survey did not have full national coverage in China, Comoros, Congo, India, Ivory Coast, and the Russian Federation. Sample sizes varied from 1,000 to 10,000 while ensuring the sample was representative of the target population. After completion of a full household roster, one adult was randomly selected per household using a Kish table [25] to be a respondent.

Fifty of the 70 WHS participating countries were classified as low and middle income economies in 2003 according to the World Bank [29]. We excluded Guatemala and Zambia because details of the complex survey design were not available in their corresponding data files; Hungary and Turkey because their questionnaires did not include all the household expenditure items; Ecuador, Nepal, Malawi, Slovak Republic and Sri Lanka because of the large extent of missing values on household expenditure items (>60%); and Zimbabwe because data on country-level out-of-pocket health expenditure was missing.

Respondents provided information on total and itemized (food, bills, education fees and supplies, health care costs excluding any insurance reimbursement, voluntary health insurance premiums or prepaid health plans, and all other goods and services) household expenditure in the past four weeks, including payments in cash and in-kind. Eight more questions were used to ascertain expenditure on hospitalization, outpatient services, traditional/alternative medicine, dentists, medications, health care products, medical tests and other services [30]. Participants were asked to exclude costs to be reimbursed by insurance and any transportation costs. The question on dentists referred to any dental procedure either for disease treatment or aesthetic reasons (excluding medications/drugs) and it was used to measure out-of-pocket expenditure for dental care in last 4 weeks.

### Variables selection

Two principal methods have been used to measure financial protection in health. Both relate a household’s out-of-pocket spending to a threshold defined in terms of living standards in the absence of the spending and are often used in parallel [31]. The first defines spending as catastrophic if it exceeds a certain percentage of the living standards measure; the second defines spending as impoverishing if it makes the difference between a household being above and below the poverty line [8]. Consistent with previous research [8, 31], CHE was present if payment for health care was ≥40% of the household capacity to pay –i.e. total household expenditure minus basic subsistence expenditure– [9, 10]. In each country, subsistence expenditure was defined as the average food expenditure of households whose food share was in the 45^th^ to 55^th^ percentile range [9, 10]. A households was considered impoverished if it fell below a relative poverty line (i.e. the subsistence expenditure derived for each country during the CHE calculation) after expenditure on health care has been subtracted from household expenditure [6, 8].

A number of covariates were included in the analysis as potential determinants of CHE and impoverishment, based on previous literature [9–17]. Sex, age, marital status and education were the individual-level factors [24]. Household characteristics included location (urban or rural), wealth index, size (number of adults and children), having a child <5 years, an adult >60 years, and insurance status (none, some and all members of the household have insurance). The calculation of the household wealth index from the WHS data has been described elsewhere [24, 32]. This index was then categorized into tertiles to enhance comparability across countries. Contextual factors were average national income (GDP per capita converted to current US dollars), income inequality (Gini coefficient expressed as percentage) and out-of-pocket health expenditure for 2003 [29] to match the mid-point of the 2002–2004 WHS data.

### Data analysis

All analyses were conducted in R software (http://www.r-project.org), using weights to produce representative estimates and incorporating survey design features (stratification and clustering) to obtain correct standard errors. The proportion of households facing CHE and impoverishment and that of households paying for dental care, including 95% confidence intervals, was reported for the full sample and each individual country. These findings were also reported for low-, lower-middle and upper-middle income countries (LIC, LMIC and UMIC, respectively).

The association of expenditure on dental care with CHE and impoverishment was evaluated using two-level logistic regression (households at level-1 and countries at level-2). Multilevel analyses were conducted on the unweighted sample as the level-2 weights, needed to compensate for the unequal probability of selection of the clusters [33, 34], were not provided in the WHS data files. To address the primary aim of the study, the association between payment for dental care and CHE was first estimated at crude level, and then adjusted for all individual- and country-level factors. This association was then estimated in stratified analysis by World Bank’s income group (based on 17 LIC, 15 LMIC and 8 UMIC, respectively), controlling for all individual- and country-level factors (GDP per capita was also included to control for residual variations between countries in a given income group). To address the second aim of the study, we added to the multilevel regression model binary indicators representing whether a household paid for each of the other seven types of health services assessed in the WHS questionnaire (inpatient care, outpatient care, traditional medicine, medications or drugs, health care products, medical tests and other health services). This fully adjusted model provided an indication of the relative contribution of payments for dental care relative to those for other health services. The same set of models was used to assess the impact of payments for dental care on impoverishment.

## Results

We analyzed data from 174,257 adults in 40 low and middle income countries with no missing values on variables of interest (62,961 in 17 LIC, 58,388 in 15 LMIC and 52,908 in 8 UMIC). The proportion of households with expenditure on dental care in the past four weeks was 7.0%, ranging from 1.5% in Myanmar and Laos to 23.7% in Russian Federation, without a clear pattern by national economic development. CHE and impoverishment were more common among poorer countries. The prevalence of CHE was 10.7%, ranging from 3.1% in Namibia to 29.8% in Bangladesh whereas the prevalence of impoverishment was 4.1%, varying from 0.5% in Czech Republic to 10.8% in Bangladesh (Table 1).

**Table 1:** Proportion of households facing catastrophic health expenditure (CHE), impoverishment and out-of-pocket payments for dental care in low, lower middle and upper middle income countries

Income group	Country	CHE		Impoverishment		Paid for dental care	
		%	(95% CI)	%	(95% CI)	%	(95% CI)
Low	Bangladesh	29.8	(8.0–31.6)	10.8	(9.6–12.1)	7.9	(6.9–9.0)
Income	Burkina Faso	12.4	(11.0–14.0)	5.3	(4.5–6.2)	1.7	(1.3–2.2)
Countries	Chad	6.6	(5.1–8.3)	3.1	(2.2–4.2)	3.3	(2.4–4.4)
	Comoros	21.1	(19.1–23.2)	10.2	(8.6–12.0)	9.4	(7.7–11.4)
	Congo, Republic	19.7	(14.1–26.3)	10.2	(6.6–14.9)	6.2	(2.9–11.2)
	Ivory Coast	14.6	(12.6–16.7)	6.9	(5.5–8.5)	4.0	(3.0–5.1)
	Ethiopia	7.1	(5.7–8.8)	3.0	(2.3–3.9)	1.8	(1.2–2.7)
	Ghana	11.5	(10.1–13.0)	4.4	(3.6–5.3)	2.4	(1.7–3.1)
	India	19.4	(17.8–21.1)	9.2	(7.9–10.5)	6.8	(5.5–8.2)
	Kenya	6.9	(5.8–8.2)	3.1	(2.4–3.9)	4.5	(3.5–5.8)
	Lao PDR	12.9	(11.3–14.6)	6.1	(5.2–7.2)	1.5	(1.1–2.0)
	Malawi	4.3	(3.5–5.2)	2.0	(1.5–2.5)	1.7	(1.3–2.3)
	Mauritania	4.8	(3.5–6.5)	2.2	(1.5–3.0)	7.5	(5.8–9.6)
	Myanmar	11.5	(10.0–13.1)	3.9	(3.3–4.6)	1.5	(1.1–2.0)
	Pakistan	23.4	(20.5–26.5)	9.6	(8.1–11.2)	10.0	(7.3–13.2)
	Senegal	9.3	(7.0–12.0)	2.9	(1.7–4.6)	10.3	(7.8–13.2)
	Vietnam	12.8	(10.2–15.7)	4.3	(3.2–5.7)	2.0	(1.1–3.4)
Lower	Bosnia & Herzegovina	9.7	(7.0–13.1)	2.5	(1.7–3.6)	14.6	(8.4–22.9)
Middle	Brazil	19.8	(18.3–21.4)	8.0	(7.0–8.9)	13.4	(12.1–14.8)
Income	China	15.6	(13.3–18.0)	5.3	(3.8–7.0)	2.5	(1.8–3.4)
Countries	Dominican Republic	8.8	(7.1–10.7)	4.4	(3.5–5.3)	5.5	(4.4–6.7)
	Georgia	11.1	(9.1–13.2)	3.5	(2.6–4.5)	12.4	(9.6–15.5)
	Kazakhstan	15.1	(11.8–18.9)	3.5	(2.4–4.9)	10.7	(8.6–13.0)
	Morocco	20.3	(18.5–22. 2)	7.6	(6.3–9.0)	8.3	(6.7–10.1)
	Namibia	3.1	(2.4–3.8)	1.2	(0.8–1.7)	2.3	(1.6–3.1)
	Paraguay	14.4	(13.2–15.7)	5.3	(4.6–6.0)	12.3	(11.1–13.7)
	Philippines	12	(10.9–13.2)	4.5	(3.9–5.2)	4.7	(3.9–5.7)
	Russian Federation	8.4	(6.2–11.0)	3.3	(2.0–5.1)	23.7	(19.8–27.9)
	South Africa	4.3	(3.3–5.6)	1.7	(1.1–2.3)	4.8	(3.3–6.9)
	Swaziland	4.4	(3.0–6.2)	1.3	(0.7–2.1)	10.2	(7.4–13.5)
	Tunisia	18.3	(16.5–20.1)	6.2	(5.3–7.2)	5.4	(4.5–6.4)
	Ukraine	20.0	(15.4–25.1)	8	(5.2–11.6)	19.6	(14.8–25.0)
Upper	Croatia	4.9	(3.4–6.8)	1.5	(0.7–2.9)	7.4	(5.4–9.7)
Middle	Czech Republic	3.4	(1.9–5.4)	0.5	(0.1–1.4)	9.3	(5.7–14.1)
Income	Estonia	11.0	(9.0–13.2)	2.8	(2.0–3.8)	16.5	(14.0–19.3)
Countries	Latvia	11.1	(8.5–14.0)	3.7	(2.1–5.8)	11.9	(9.2–14.9)
	Malaysia	4.1	(3.4–4.9)	1.6	(1.2–2.0)	6.1	(5.4–7.0)
	Mauritius	11.6	(10.0–13.3)	1.9	(1.4–2.5)	5.5	(4.5–6.6)
	Mexico	5.8	(5.3–6.3)	2.3	(2.0–2.5)	11.3	(10.7–11.9)
	Uruguay	6.0	(4.1–8.2)	0.9	(0.7–1.2)	9.6	(7.8–11.7)

Households that paid for dental care had 1.88 (95% CI: 1.78–1.99) and 1.65 (95% CI: 1.52–1.80) greater odds of facing CHE and impoverishment, respectively, than those that did not pay for dental care, after controlling for all individual— and country-level covariates. An inverted V-shaped trend was noted in stratified analysis by World Bank’s income group. The odds of CHE were 1.52 (95% CI: 1.37–1.68), 2.34 (95% CI: 2.16–2.53) and 1.63 (95% CI: 1.45–1.83), and the odds of impoverishment were 1.38 (95% CI: 1.19–1.60), 1.90 (95% CI: 1.68–2.15) and 1.59 (95% CI: 1.31–1.92) in LIC, LMIC and UMIC, respectively. Older and less educated adults, poorer households, those in rural areas and having a child <5 years old, an adult >60 years old and no health insurance also have greater odds of facing CHE and impoverishment. At country level, higher out-of-pocket health expenditure was the only factor associated with greater odds of both CHE and impoverishment (Table 2).

**Table 2:** Odds ratios (95% confidence interval) of catastrophic health expenditure (CHE) and impoverishment by covariates in 40 low and middle income countries

Factors	CHE		Impoverishment	
	COR [95% CI]	AOR [95% CI]	COR [95% CI]	AOR [95% CI]
Fixed effects: Individual Level				
Paid for dental care in last 4 weeks				
No	1.00	1.00	1.00	1.00
Yes	1.81 [1.72–1.91]***	1.88 [1.78–1.99]***	1.53 [1.41–1.65]***	1.65 [1.52–1.80]***
Insurance status				
None	1.00	1.00	1.00	1.00
Some	1.02 [0.96–1.07]	0.99 [0.94–1.06]	0.79 [0.73–0.87]***	0.87 [0.78–0.96]**
All	0.80 [0.76–0.85]***	0.80 [0.75–0.85]***	0.62 [0.56–0.70]***	0.71 [0.64–0.78]***
Sex				
Women	1.00	1.00	1.00	1.00
Men	0.96 [0.93–0.99]**	0.97 [0.94–1.00]	0.94 [0.89–0.98]*	0.97 [0.92–1.02]
Age				
18–29 years	1.00	1.00	1.00	1.00
30–39 years	0.97 [0.93–1.01]	0.96 [0.91–1.00]	0.96 [0.91–1.00]	0.95 [0.89–1.02]
40–49 years	0.92 [0.87–0.96]*	0.94 [0.89–0.99]*	0.92 [0.86–0.99]*	0.97 [0.89–1.05]
50–59 years	1.04 [0.99–1.09]	1.06 [0.99–1.12]	1.04 [0.99–1.14]	1.08 [0.99–1.18]
60–69 years	1.26 [1.19–1.33]	1.04 [0.96–1.12]	1.25 [1.15–1.36]***	1.08 [0.96–1.21]
70+ years	1.18 [1.08–1.28]***	1.18 [1.08–1.28]***	1.35 [1.23–1.48]***	1.18 [1.04–1.33]**
Marital Status				
Married	1.00	1.00	1.00	1.00
Never married	0.93 [0.88–0.98]**	0.93 [0.88–0.98]**	0.87 [0.81–0.92]***	0.98 [0.90–1.06]
Previously married	0.97 [0.93–1.02]	0.97 [0.93–1.02]	1.06 [0.99–1.13]	0.99 [0.93–1.07]
Education				
Primary school	1.00	1.00	1.00	1.00
Secondary school	0.93 [0.90–0.97]	1.00 [0.96–1.04]	0.72 [0.68–0.77]***	0.86 [0.80–0.92]***
College and above	0.75 [0.71–0.80]***	0.79 [0.74–0.85]***	0.47 [0.43–0.54]***	0.62 [0.55–0.70]***
Household wealth				
First tertile (Poorest)	1.00	1.00	1.00	1.00
Second tertile (Middle)	1.11 [1.07–1.15]***	1.12 [1.08–1.17]***	0.98 [0.92–1.04]	1.02 [0.96–1.08]
Third tertile (Wealthiest)	1.07 [1.02–1.10]***	1.08 [1.04–1.13]***	0.81 [0.76–0.86]***	0.89 [0.84–0.94]***
Household size				
1–2 members	1.00	1.00	1.00	1.00
3–5 members	0.95 [0.91–0.99]*	0.93 [0.89–0.98]**	1.01 [0.94–1.07]	0.99 [0.92–1.08]
> 5 members	1.00 [0.96–1.06]	0.89 [0.84–0.95]***	1.11 [1.03–1.20]**	0.98 [0.90–1.08]
Have child <5 years				
No	1.00	1.00	1.00	1.00
Yes	1.15 [1.11–1.19]***	1.25 [1.21–1.30]***	1.28 [1.22–1.37]***	1.34 [1.26–1.42]***
Have adult >60 years				
No	1.00	1.00	1.00	1.00
Yes	1.38 [1.33–1.42]***	1.34 [1.28–1.40]***	1.30 [1.24–1.40]***	1.26 [1.17–1.34]***
Urban/rural status				
Urban	1.00	1.00	1.00	1.00
Rural	1.06 [1.02–1.09]**	1.02 [0.99–1.06]	1.46 [1.39–1.54]***	1.31 [1.24–1.38]***
Fixed effects: Country Level				
GDP per capita (1000-increase)	0.91 [0.84–0.99]*	0.92 [0.86–1.00]	0.85 [0.78–0.93]***	0.92 [0.85–0.99]*
Gini index (1-percent increase)	0.99 [0.98–1.01]	1.00 [0.98–1.02]	0.99 [0.98–1.01]	1.00 [0.98–1.02]
Out-of-pocket health expenditure	1.02 [1.01–1.02]***	1.02 [1.01–1.03]***	1.02 [1.01–1.02]***	1.02 [1.01–1.03]***

Two-level logistic regression was fitted. COR: Crude odds ratio; AOR: adjusted odds ratio

* < 0.05; ** < 0.01; *** < 0.001

Results remained unchanged after further adjustment for other types of health services used in the last 4 weeks (Table 3). Households paying for any type of health service had greater odds of facing CHE and impoverishment. However, services could be grouped depending on their impact on households. While health services in the first group (medications, hospitalization, medical tests and outpatient services) were associated with 2.57–4.13 greater odds of CHE and impoverishment, those in the second group (dental services, traditional medicine, health care products and other services) were associated with 1.53–1.92 greater odds only.

**Table 3:** Odds ratios (95% confidence interval) of catastrophic health expenditure (CHE) and impoverishment by type of health services in 40 low and middle income countries

Type of health services	% use	CHE^a^	Impoverisment^a^
Dental care	7.0	1.88 [1.78-1.99]***	1.88 [1.78-1.99]***
Inpatient care	4.2	3.93 [3.70-4.17]***	3.24 [2.99-3.53]***
Outpatient care	14.1	3.11 [3.01-3.31]***	2.57 [2.47-2.73]***
Traditional medicine	6.1	1.82 [1.72-1.92]***	1.81 [1.67-1.96]***
Medications or drugs	42.7	4.13 [3.97-4.28]***	3.49 [3.29-3.70]***
Health care products	3.5	1.88 [1.75-2.02]***	1.76 [1.56-1.95]***
Medical tests	6.3	3.80 [3.62-3.99]***	2.88 [2.68-3.09]***
Other health services	4.1	1.92 [1.80-2.05]***	1.53 [1.38-1.69]***

^a^Models included all services listed in the table plus all individual-level (sex, age, marital status, education, household wealth, household size, have child <5 years, have adult >60 years, urban/rural status and insurance status) and country-level factors (GDP per capita, Gini index, out-of-pocket health expenditure) as explanatory variables

***p<0.001

## Discussion

This study shows that out-of-pocket payments for dental care can pose a considerable burden on households in low and middle income countries, to the extent of preventing expenditure on basic necessities and pushing families into poverty. Our findings also show that the impact of paying for dental care was similar to that of traditional medicine, health care products and other services but lower than that of medications, hospitalizations, medical tests and outpatient care.

Some study limitations need to be addressed. First, the WHS data are over a decade old. However, we know of no other internationally comparable data for low and middle income countries. Only 5 of the 40 countries evaluated (Croatia, Czech Republic, Estonia, Latvia and Uruguay) are now classified as high-income economies according to the latest World Bank’s ranking, supporting the relevance of our findings for most of the countries evaluated. In addition, the few countries with national data post-2010, like Brazil [35], China [12] and Ghana [36], showed similar estimates for CHE and impoverishment to those reported in this analysis. Second, our analysis was based on cross-sectional data, and thus, unable to test for causal relationships. Although longitudinal panel data would be preferable to estimate the impact of spending on health care on living standards, often only cross-sectional data are available. Third, our expenditure estimates were derived from 14 items with a recall frame of 4 weeks. It has been previously shown that estimates of health expenditure are affected by the methodology used to collect data [37–39]. However, the WHS expenditure data had good test-retest reliability [30]. Fourth, spending on dental care was one of eight items used to gather information on households’ health expenditure. It is thus possible that some overlap between the 8 types of health services existed; for instance, those related to the use of drugs and/or traditional medicine for dental pain relief or children receiving dental treatment under general anesthesia in hospital settings. Therefore, our estimates may be somewhat conservative.

Our findings suggest that households were more likely to reduce spending on other goods and services, and be impoverished, disrupting their living standards, because a household member needed to use and pay for dental services. Although the impact of out-of-pocket expenses for dental care was noted across all World Bank’s income groups, an inverted V-shaped trend was identified by economic development. That is, a stronger impact of payments for dental care was found among LMIC than in LIC and UMIC. This trend may be explained by a combination of factors affecting countries as they move through the different stages of economic development. The impact of payments for dental care in LIC may be underestimated because people with treatment needs cannot afford the costs of dental services [24] or the lack of dentists to provide appropriate treatment (dental services would then be rationed). The inverted V-shaped trend may thus represent those who can meet the costs to access needed dental care services. In LMIC, there will be more supply of private dental care providers and more perceived need for care which could increase the risk of households facing CHE and impoverishment. Households in UMIC may have expenditures than in other income groups but they are not catastrophic given greater economic resources in households, greater supply of dental providers and/or some level of public offer. Aside from economic development, we also found that countries with greater levels of out-of-pocket health expenditure also have more households facing CHE and impoverishment [9]. Our findings also corroborate previous evidence characterizing households more likely to face the financial consequences of paying for health care [11–17]. Poorer and rural households and those with young children, older adults and no health insurance were more likely to face CHE and impoverishment.

All types of health services had a financial impact on households. However, there was a clear separation between health services, which was not related to the typical split between inpatient vs. outpatient care. Indeed, buying medication over the counter had a similar effect as a hospital admission. Our findings also indicate that the impact of paying for dental care was lower than paying for medications, hospitalizations, diagnostics and outpatient care, but similar to that of traditional care, health care products (such as prescription glasses, hearing aids, prosthetic devices, etc.) and other health care services that were not included above. It is worth mentioning that the proportion of the population using dental services was smaller than that using other types of health services (especially medications and outpatient care), which highlights two interrelated possibilities; the lower availability of dental services, on one hand, and the fact that dental costs per session may be too high so as to cause financial shocks to families, on the other.

Looking forward, there are several policy options worth exploring to help reduce the economic burden caused by oral conditions. Oral and non-communicable diseases (NCDs) have shared determinants, and as such, can be addressed by common organized efforts. Regulating the tobacco and sugar industry can reduce the risks of several NCDs, including dental caries and periodontal disease. Beyond prevention, our results indicate that developing countries need to explore strategies to improve financial protection in the dental care sector. Alternative financing mechanisms for risk pooling and prepayments may be an sustainable option, although the status quo dental insurance model in developed countries still leads to affordability issues for consumers [23]. The dental profession has the responsibility to advocate for the inclusion of dental care in ongoing debates on universal health coverage [24]. Universal health coverage and insurance schemes should cover both prevention and control of oral diseases. In terms of research, it would be interesting to corroborate our findings with more recent data, even if that implies using a smaller sample of countries. Further studies could also expand on this area by looking into the mechanisms families use to cope with out-of-pocket expenditures for dental care. Given the dual nature of dental care, there is also a need for evaluating the impact of specific dental treatments, particularly those that are considered essential (disease treatment) and cosmetic/aesthetic.

## Conclusion

This study provides evidence of the financial burden that out-of-pocket expenditure for dental care put on households in low and middle income countries. Our findings indicate that out-of-pocket spending on dental care contributes to catastrophic health spending that can push households into poverty. Current financing mechanisms for dental care in developing countries do not protect their citizens from the financial impact of oral conditions. Dental care financing reforms ought to be considered, including integrating the prevention and control of oral diseases into universal health insurance coverage programs.

## Abbreviations

CHE: Catastrophic health expenditure
LIC: Low income countries
LMIC: Lower middle income countries
NCD: Non-Communicable diseases
UMIC: Upper middle income countries
WHS: World health surveys

## References

[1] WHO (2005). Sustainable health financing, universal coverage and social health insurance. Resolution WHA58.33.

[2] WHO (2010). World Health Report 2010. Health systems financing: the path to universal coverage.

[3] McIntyre D, Ranson MK, Aulakh BK, Honda A (2013). Promoting universal financial protection: evidence from seven low- and middle-income countries on factors facilitating or hindering progress. Health Res Policy and Systems/BMC.

[4] Reich MR, Harris J, Ikegami N, Maeda A, Cashin C, Araujo EC, Takemi K, Evans TG (2016). Moving towards universal health coverage: lessons from 11 country studies. Lancet.

[5] Jaspers L, Colpani V, Chaker L, van der Lee SJ, Muka T, Imo D, Mendis S, Chowdhury R, Bramer WM, Falla A (2015). The global impact of non-communicable diseases on households and impoverishment: a systematic review. Eur J Epidemiol.

[6] Wagstaff A, van Doorslaer E (2003). Catastrophe and impoverishment in paying for health care: with applications to Vietnam 1993–1998. Health Econ.

[7] McIntyre D, Thiede M, Dahlgren G, Whitehead M (2006). What are the economic consequences for households of illness and of paying for health care in low- and middle-income country contexts?. Soc Sci Med.

[8] Wagstaff A. Measuring financial protection in health. Policy Research Working Paper Series 4554. Washington DC: The World Bank; 2008.

[9] Xu K, Evans DB, Kawabata K, Zeramdini R, Klavus J, Murray CJ (2003). Household catastrophic health expenditure: a multicountry analysis. Lancet.

[10] Xu K, Evans DB, Carrin G, Aguilar-Rivera AM, Musgrove P, Evans T (2007). Protecting households from catastrophic health spending. Health Aff (Millwood).

[11] Knaul FM, Wong R, Arreola-Ornelas H, Mendez O (2011). Household catastrophic health expenditures: a comparative analysis of twelve Latin American and Caribbean countries. Salud Publica Mex.

[12] Li Y, Wu Q, Xu L, Legge D, Hao Y, Gao L, Ning N, Wan G (2012). Factors affecting catastrophic health expenditure and impoverishment from medical expenses in China: policy implications of universal health insurance. Bull World Health Organ.

[13] Van Minh H, Kim Phuong NT, Saksena P, James CD, Xu K (2013). Financial burden of household out-of pocket health expenditure in Viet Nam: findings from the national living standard survey 2002–2010. Soc Sci Med.

[14] Limwattananon S, Tangcharoensathien V, Prakongsai P (2007). Catastrophic and poverty impacts of health payments: results from national household surveys in Thailand. Bull World Health Organ.

[15] Kim Y, Yang B (2011). Relationship between catastrophic health expenditures and household incomes and expenditure patterns in South Korea. Health policy.

[16] Kavosi Z, Rashidian A, Pourreza A, Majdzadeh R, Pourmalek F, Hosseinpour AR, Mohammad K, Arab M (2012). Inequality in household catastrophic health care expenditure in a low-income society of Iran. Health Policy Plan.

[17] Ozgen Narci H, Sahin I, Yildirim HH (2015). Financial catastrophe and poverty impacts of out-of-pocket health payments in Turkey. Eur J Health Econ.

[18] Kassebaum NJ, Bernabé E, Dahiya M, Bhandari B, Murray CJ, Marcenes W (2015). Global burden of untreated caries: a systematic review and metaregression. J Dent Res.

[19] Kassebaum NJ, Bernabé E, Dahiya M, Bhandari B, Murray CJ, Marcenes W (2014). Global burden of severe periodontitis in 1990–2010: a systematic review and meta-regression. J Dent Res.

[20] Kassebaum NJ, Bernabé E, Dahiya M, Bhandari B, Murray CJ, Marcenes W (2014). Global burden of severe tooth loss: a systematic review and meta-analysis. J Dent Res.

[21] Listl S, Galloway J, Mossey PA, Marcenes W (2015). Global economic impact of dental diseases. J Dent Res.

[22] Vujicic M, Bernabé E, Neumann DG, Quinonez C, Mertz E, Scheffler RM (2016). Dental care. World scientific handbook of global health economics and public policy (volume 2 - health determinants and outcomes).

[23] Vujicic M (2014). How affordable is health care in the United States and other countries?. J American Dental Assoc.

[24] Masood M, Sheiham A, Bernabé E (2015). Household expenditure for dental care in low and middle income countries. PLoS One.

[25] Ustun TB, Chatterji S, Mechbal A, Murray CJL, Murray CJL, Evans D (2003). The world health surveys. Health systems performance assessment debates, methods and empiricism.

[26] Moussavi S, Chatterji S, Verdes E, Tandon A, Patel V, Ustun B (2007). Depression, chronic diseases, and decrements in health: results from the World Health Surveys. Lancet.

[27] Obermeyer Z, Murray CJ, Gakidou E (2008). Fifty years of violent war deaths from Vietnam to Bosnia: analysis of data from the world health survey programme. BMJ (Clinical research ed).

[28] Loerbroks A, Herr RM, Subramanian S, Bosch JA (2012). The association of asthma and wheezing with major depressive episodes: an analysis of 245 727 women and men from 57 countries. Int J Epidemiol.

[29] World Bank (2005). World development indicators.

[30] Xu K, Ravndal F, Evans DB, Carrin G (2009). Assessing the reliability of household expenditure data: results of the World Health Survey. Health policy.

[31] O’Donnell O, van Doorslaer E, Wagstaff A, Lindelow M (2008). Analysing health equity using household survey data: a guide to techniques and their implementation. In.

[32] Bhandari B, Newton JT, Bernabé E (2015). Income inequality and use of dental services in 66 countries. J Dent Res.

[33] Carle AC (2009). Fitting multilevel models in complex survey data with design weights: Recommendations. BMC Med Res Methodol.

[34] Cai TJ (2013). Investigation of ways to handle sampling weights for multilevel model analyses. Sociol Methodol.

[35] Boing AC, Bertoldi AD, Posenato LG, Peres KG (2014). The influence of health expenditures on household impoverishment in Brazil. Rev Saude Publica.

[36] Aryeetey GC, Westeneng J, Spaan E, Jehu-Appiah C, Agyepong IA, Baltussen R (2016). Can health insurance protect against out-of-pocket and catastrophic expenditures and also support poverty reduction? evidence from Ghana’s national health insurance scheme. Int J Equity Health.

[37] Lu C, Chin B, Li G, Murray CJ (2009). Limitations of methods for measuring out-of-pocket and catastrophic private health expenditures. Bull World Health Organ.

[38] Lavado RF, Brooks BP, Hanlon M (2013). Estimating health expenditure shares from household surveys. Bull World Health Organ.

[39] Raban MZ, Dandona R, Dandona L (2013). Variations in catastrophic health expenditure estimates from household surveys in India. Bull World Health Organ.

